# Fibronectin within Sodium Alginate Microcapsules Improved Osteogenic Differentiation of BMMSCs in Dose Dependent Manner by Targeting SP7, OCN, CDK1, ZBTB16, and Twist1 Expression

**DOI:** 10.34172/apb.2022.012

**Published:** 2021-08-07

**Authors:** Karim Shamsasenjan, Younes Beygi Khosrowshahi, Mahsa Mahmoodi, Parvin Akbarzadehlaleh, Nesrin Gareayaghi, Babak Nejati

**Affiliations:** ^1^Immunology Research Center, Tabriz University of Medical Sciences, Tabriz, Iran.; ^2^Stem Cell and Tissue Engineering Research Laboratory, Azerbaijan Shahid Madani University, Tabriz, Iran.; ^3^Department of Pharmaceutical Biotechnology, Faculty of Pharmacy, Tabriz University of Medical Sciences, Tabriz, Iran.; ^4^Istanbul Sisli Hamidiye Etfal Training and Research Hospital, Blood Center, University of Health Science, Istanbul, Turkey.; ^5^Hematology and Oncology Research Center, Tabriz University of Medical Sciences, Tabriz, Iran.

**Keywords:** Bone marrow-derived MSCs (BMMSCs), Alginate, Fibronectin, Microcapsules, osteogenic differentiation

## Abstract

***Purpose:*** Insoluble fibronectin as an extracellular matrix (ECM) protein has the potential to promote proliferation, differentiation, and migration of mesenchymal stem cells (MSCs). However, there is limited information about the effects of fibronectin various concentrations on bone marrow-derived MSCs (BMMSCs) function and differentiation.

***Methods:*** In this experimental study, using a gel injection device, BMMSCs were encapsulated in sodium alginate microcapsules containing 1.25% alginate, 1% gelatin, and fibronectin (0.01, 0.05, 0.1, and 0.2 μg/ml). MTT assay was used to examine the proliferation of BMMSCs. Also, BMMSCs apoptosis were analyzed using Annexin-V/PI staining and fluorescence activated cell sorting (FACS). Alkaline phosphatase (ALP) test was conducted to assess BMMSCs osteogenic differentiation potential. Finally, mRNA expression levels of the SP7, osteocalcin (OCN), Twist Family BHLH Transcription Factor 1 (Twist1), Peroxisome proliferator‐activated receptor γ^2^ (PPARγ^2^), Cyclin-dependent kinase 1 (CDK1), and Zinc Finger and BTB Domain Containing 16 (ZBTB16), following exposure with fibronectin 0.1 μg/ml.

***Results:*** According to results, fibronectin had the potential to promote proliferation rates of the BMMSCs, in particular at 0.1 and 0.2 μg/ml concentrations. we showed that the fibronectin was not able to modify apoptosis rates of the BMMSCs. ALP test results approved the notable potential of the fibronectin, to trigger osteogenic differentiation of the BMMSCs. Also, RT-PCR results indicated that fibronectin 0.1 μg/ml could augment osteogenic differentiation of cultured BMMSCs through targeting of OCN, SP7, Twist1, CDK1, and ZBTB16, strongly or slightly.

***Conclusion:*** Results showed that fibronectin can improve proliferation and osteogenic differentiation of BMMSCs without any effect on these cells' survival.

## Introduction

According to the wide range of studies, the cells should make contact with other cells and extracellular matrix (ECM) to become biologically functional.^1^ The ECM is a three-dimensional (3D) network that plays key roles in supporting the physical and also biological structure and function of the living cells.^2^ As cells in the body perform their function within a complex 3D environment that cannot be formed by two-dimensional (2D) medium and animal models, 3D culture media have been used widely in medical science and tissue engineering. The most pivotal properties of the 3D culture media are keeping of homeostasis for a long time, the transmission of nutrients and oxygen by microfluidic systems and also the creation of the barrier tissue and integration of the flow.^3,4^ Microencapsulation of cells is an interesting technique of the entrapping of the living cells within a semi-permeable matrix that provides a 3D culture medium for cells.^5^ Alginate is a natural anionic polysaccharide formed by several residues of d-mannuronate and l-guluronate with (1-4) linkages and is a biomaterial commonly applied as a defensive barrier.^6,7^ Based on the analysis, the connection of sodium cation to alginate chains makes a gelatin structure associated microencapsulated cell viabilities.^8^ Due to the lack of toxicity and high bioavailability, sodium alginate microcapsules possess a noticeable potential to encapsulate mesenchymal stem cells (MSCs).^9^ MSCs are non-hematopoietic multipotent cells found in varied types of the tissues, in particular, bone marrow and adipose tissue.^10^ They are spindle-shaped cells possessed self-renewal ability and a great potential to differentiate into adipocyte, chondrocyte, and osteoblast cells.^11^ Bone marrow MSCs (BMMSCs) are positive for CD73, CD90, CD 22, and CD105 but negative for CD11b, CD14, CD19, CD34, CD45, CD79a, and HLA-DR.^12,13^ Because of the easy isolation and expansion in the laboratory and also the immunomodulatory effect on innate and adaptive immune systems, BMMSCs are considered the best types of stem cells for culture in 3D media to use for therapeutic applications.^14^

Fibronectin is a high molecular weight glycoprotein found insoluble, within the plasma, as well as insoluble forms.^15^ The insoluble form is part of the ECM, which promotes MSCs growth and resistance to apoptosis via targeting of the cell cycle.^16,17^ Regarding evidence, in bone tissue, fibronectin is made by osteoblast cells and exerts its effects by connecting to integrin molecules on BMMSCs surface and induction of Runt-related transcription factor-2 (RUNX2) signaling pathway, which is mediated by fibronectin RGD (Arg-Gly-Asp)-containing peptide.^18^ Based on molecular studies, RUNX2 signaling, which involved in osteogenic differentiation of MSCs, regulates varied types of the molecular signaling pathways, including BMP, Wnt, Hedgehog, and Notch, directly or indirectly.^19,20^ According to experiments conducted on rat calvaria, fibronectin is increased in periosteum and osteoid around the localized implant^21^; in fact, fibronectin is augmented in part of bone tissue where MSC become committed, and in turn, recruited to osteoblast cells.^22^ Considering the results obtained from available evidence, fibronectin promotes MSCs activities, but there is limited data about the fibronectin’s various concentrations effects on MSCs biology and function. In this study, we investigated the effects of the different concentrations of fibronectin on proliferation, differentiation, and apoptosis of BMMSCs. Furthermore, we evaluated the expression levels of some genes that have a negative or positive role in osteogenic differentiation of the BMMSCs. Our findings lead to the optimization of BMMSC proliferation in a laboratory environment as well as the treatment of bone diseases.

## Materials and Methods

### Cell culture

In this experimental study, BMMSCs were purchased from Bonyakteh Research Center, Tehran, Iran and cultured in 50-cm^2^ filtered flask within Dulbecco’s Modified Eagle Medium (DMEM)-LG (Gibco, UK) supplemented with 10% fetal bovine serum (FBS, Gibco, UK) and 0.1% penicillin/streptomycin (Gibco, UK) without any additional growth factors and incubated at 37°C with 5% CO_2_ (N-Biotek NB203xl). When the cells reached 80-90% confluence, MSCs were trypsinized and passaged, and the experiments were conducted with MSCs from the fourth passage.

### Microencapsulation

Encapsulation of MSCs into small (200-µm) sodium alginate microcapsules was conducted as described elsewhere.^23^ Briefly, under sterile conditions, the cells were mixed with 1.25% (w/v) sodium alginate solution (Sigma-Aldrich, USA) and 2% (w/v) gel solution (Sigma-Aldrich, USA) as well as four different concentration of fibronectin (0.05, 0.01, 0.1, and 0.2 µg/mL) to obtain a alginate/cell/fibronectin/gel mixture with final density of 1.5×10^6^ cells.

The mixture was dripped into 100mM CaCl_2_ solution using electrostatic microbead (microcapsule) generator in 9 volts to obtain plain alginate microbeads, which were then collected by brief centrifugation (500 rpm for 3 min) and three times washed by CF-KRH. To induce differentiation, the beads were cultured in conventional osteogenic medium containing maintenance medium supplemented with 50 mg ascorbic-acid-2 phosphate (Sigma-Aldrich, USA), 0.1mg dexamethasone (Sigma-Aldrich, USA), and 10mM B-glycerophosphate (Sigma-Aldrich, USA) and incubated at 37°C with 5% CO_2_ for 21 days, and the medium was changed every three days.

### MTT assay

The proliferation of BMMSCs following exposure with fibronectin was assessed using MTT assay. To investigate the proliferation of BMMSCs encapsulated in sodium alginate microcapsules, the microcapsules were seeded in a maintenance medium containing DMEM/LG plus 10% FBS and 1% penicillin/streptomycin and incubated in 37°C with 5% CO_2_. The MTT test was performed within 1-10 days of exposure with 0.01, 0.05, 0.1 and 0.2 μg/mL concentration of fibronectin. For MTT assay, the beads were transferred into 24-well plates (Falcon, USA), 100 µl of MTT solution was added to each well and incubated at 37°C with 5% CO_2_ for 4 hours. Then, 1ml of DMSO (Invitrogen, USA) was added to each well of the plate and shaken for 20 minutes. The absorbance of solutions was recorded at 490 nm by a spectrophotometer^24^ (UV 2100, USA).

### Detection of apoptosis by flow cytometry

To investigate apoptosis of BMMSCs encapsulated in sodium alginate microcapsules, the microcapsules were seeded in a maintenance medium containing DMEM/LG plus 10% FBS and 1% penicillin/streptomycin and incubated in 37°C with 5% CO^2^. Apoptosis of BMMSCs was calculated using the Annexin V-FITC Apoptosis Detection Kit (BD Pharmingen, USA). Accordingly, 48 hours after exposure to various concentrations of fibronectin, BMMSCs were washed with PBS and resuspended in 100 μL annexin V-FITC labeling solution. Then cells exposed with 5 μL annexin V-FITC and 5 μL propidium iodide (PI) for 30 minutes at room temperature and in the dark condition.^25^ Finally, cells were evaluated using FACSCalibur (BD Biosciences, Franklin Lakes, NJ) and results were analyzed by FlowJo V. 10 software.

### Alizarin-red staining

For confirmation of the osteoblastic differentiation of human BMMSCs cells, Alizarin-red staining was conducted. Accordingly, cells were fixed with 10% formalin for 15 min and stained with 1ml of 1% alizarin red solution in water (pH 4.2) at for 20 minutes at 25°C after 10,15 and 21 days.^26^ Finally, the stained matrix was observed under an inverted microscope with several magnifications.

### Quantitative real-time polymerase chain reaction

The total RNA of BMMSCs and osteoblasts was extracted on Days 0, 5, 10, 15 and 21 using the TRIzol reagent (Sigma-Aldrich, Darmstadt, Germany) according to the manufacturers’ protocol. The validity of the yields was evaluated by electrophoresis and calculated by Nanodrop. Then mRNAs were reverse‐transcribed to the complementary DNAs (cDNAs) by cDNA synthesis Kit (Takara, Tokyo, Japan).^11^ The Real‐time PCR was performed by Corbett machine using the SYBR Green reagent (Thermo Fisher Scientific, Waltham, MA). All primer pair sequences for experimented genes are cited in Table 1.

**Table 1:** List of primer pairs used for quantitative PCR (qPCR)

**Gene**		**Primer sequences**	**Product Size (bp)**
RUNX2	F	CGCCTCACAAACAACCACAG	225
	R	TCACTGTGCTGAAGAGGCTG	225
BSP	F	AAGGGCACCTCGAAGACAAC	119
	R	CCCTCGTATTCAACGGTGGT	119
BMP-2	F	ACTCGAAATTCCCCGTGACC	144
	R	CCACTTCCACCACGAATCCA	144
OCN	F	TCCTTTGGGGTTTGGCCTAC	148
	R	CCAGCCTCCAGCACTGTTTA	148
Twist1	F	GCCAGGTACATCGACTTCCTCT	122
	R	TCCATCCTCCAGACCGAGAAG	122
PPARγ2	F	ATGCACTGCCTATGAGCACT	147
	R	CAACTGTGGTAAAGGGCTTG	147
SP7	F	AGTCAGAGTAGGACTGTAGGAC	247
	R	AGTGAACTTCCTCCTCAAGC	247
CDK1	F	TGGAGAAGGTACCTATGGAGTTG	178
	R	AGGAACCCCTTCCTCTTCAC	178
ZBTB16	F	GAGCTTCCTGATAACGAGGCTG	107
	R	AGCCGCAAACTATCCAGGAACC	107
GAPDH	F	GAGTCAACGGATTTGGTCGT	196
	R	TTGATTTTGGAGGGATCTCG	196

***Note***. RUNX2; Runt-related transcription factor 2, BSP; Bone sialoprotein, BMP-2; Bone morphogenetic protein 2, OCN; Osteocalcin, Twist1; Twist Family BHLH Transcription Factor 1, PPARγ2; Peroxisome proliferator‐activated receptor γ2, CDK1; Cyclin-dependent kinase 1, ZBTB16; Zinc Finger And BTB Domain Containing 16, GAPDH; Glyceraldehyde-3-phosphate dehydrogenase.

### Alkaline phosphatase (ALP) test

Osteogenic differentiation of BMMSCs was assessed by the ALP test (Sigma- Aldrich, USA) within 5, 10, 15, and 21 days post-induction of differentiation. To measure ALP activity, the microcapsules were transferred into 24-well plates, fixed with paraformaldehyde and washed; then, 100 µL of para-nitrophenylphosphate (Pnpp) solution was added to each well of the plate, and the absorption was recorded at 450nm using a spectrophotometer.^11^

### Statistical analysis

Statistical analyses were performed using GraphPad Prism version 8.01. A one-way ANOVA test was used for determining the statistically significant differences. All results were expressed as the mean ± SEM from three independent experiments. Also, *P* values<0.05 were noticed statistically significant.

## Results

### Confirming of the BMMSCs osteoblastic differentiation by Alizarin‐redstaining

**Figure 1:**
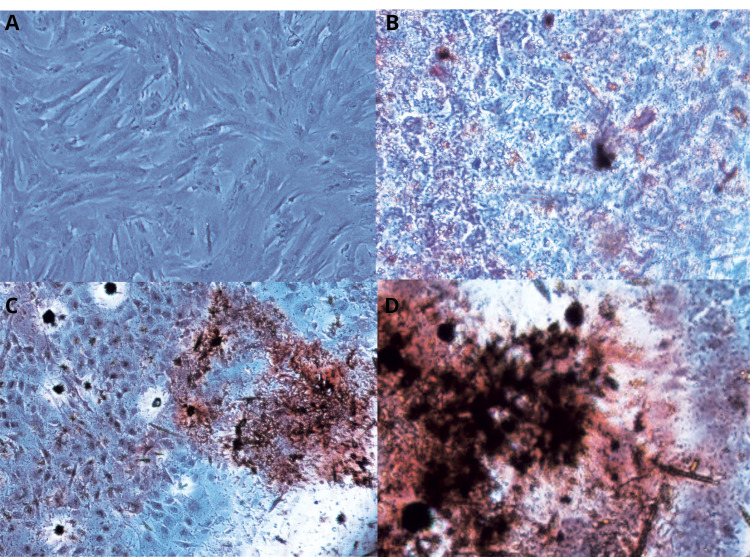
**Confirming of the BMMSCs osteoblastic differentiation by Alizarin‐red staining** :Post-induction of Osteogenic differentiation BMMSCs was stained by Alizarin‐red in day 0, 10,15 and 21.

The osteoblastic differentiation of the BMMSCs was approved by absorption of the Alizarin‐red stain in ECM of the BMMSCs because of the occurring of the calcium mineralization on 10, 15 and 21days of cell cultures (Figure 1).

### Confirming of the BMMSCs osteoblastic differentiation by evaluation of the mRNA expression levels of SP7, BMP2, BSP, and RUNX2

**Figure 2:**
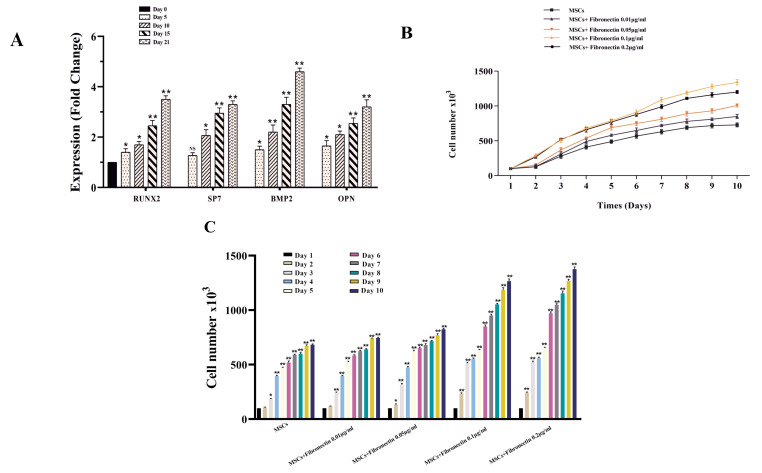
**mRNA expression of the osteoblastic marker genes after osteogenic differentiation, SP7, BMP2, BSP, and RUNX2 by Real-Time PCR**: fig 2A shows the fold change of SP7, BMP2, BSP, and RUNX2 genes expression in day 0, 10,15 and 21 Post-induction of Osteogenic differentiation. Fig2B displays effect of different fibronectin concentration in cell medium on expansion using MTT assay. Fig3: BMMSCs number changes in different concentration of fibronectin were statically analyzed in day 0 to 10. (*P* value =0.05)

Osteoblastic differentiation of the BMMSCs was approved by assessment of the mRNA expression of the osteoblastic marker genes, such as SP7, BMP2, BSP, and RUNX2 by Real-Time PCR (Figure 2A). Based on the results, the mRNA levels were strongly promoted over time progression in differentiation‐induced MSCs (*P* < 0.05) (Figure 2A). The mRNA expression levels of the candidate genes were meaningfully higher than compared with other periods (*P* < 0.05) (Figure 2A).

### Fibronectin augmented proliferation potential of the BMMSCs

Based on results, 0.01-0.2 μg/mL of fibronectin were able to enhance proliferation rates of the exposed BMMSCs compared with the control cells, in particular, 5-10 days after exposure (Figure 2B, C). According to the analysis, the effects of fibronectin 0.1 μg/mL on BMMSCs proliferation were more prominent than other concentrations (Figure 2B, C). Surprisingly, fibronectin 0.1 μg/mL had the potential to enhance the proliferation of target cells more powerful than fibronectin 0.2 μg/mL (Figure 2B, C).

### Fibronectin had not any effect on BMMSCs apoptosis

**Figure 3:**
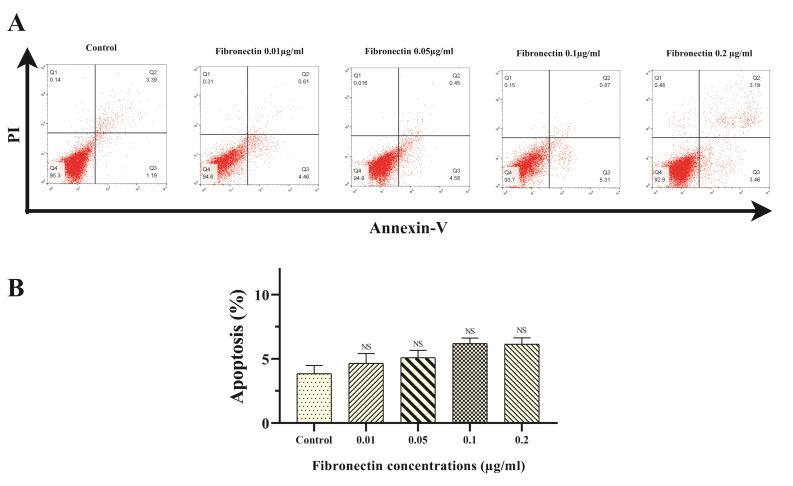
**Apoptosis rates of BMMSCs-exposed with fibronectin 0.01, 0.05, 0.1 and 0.2 μg/ml in comparison to control cells Annexin-V/PI staining and FACS analysis (** *P* **<0.05**). Fig3A BMMSCs was cultured for 48 hours in presence c, stained with Annexin-V/PI and analyzed by flow cytometry. Fig3B The comparison fibronectin treated BMMSCs’ apoptosis rate (*P* value =0.05)

There was no significant shift in apoptosis rates of BMMSCs-exposed with fibronectin 0.01, 0.05, 0.1 and 0.2 μg/mL in comparison to control cells (untreated BMMSCs cells) at 48 hours of exposure measured by Annexin-V/PI staining and FACS analysis (*P* < 0.05) (Figure 3A, B). Based analysis, apoptosis percentages in control, cells treated with fibronectin 0.01, 0.05, 0.1 and 0.2 μg/mL were 4.14±1.98, 4.92±2.15, 5.11±1.78, 6.03±1.33 and 7.16±1.73% of total cells, respectively (Figures 3A, B).

### Fibronectin improved osteoblastic differentiation of BMMSCs

**Figure 4:**
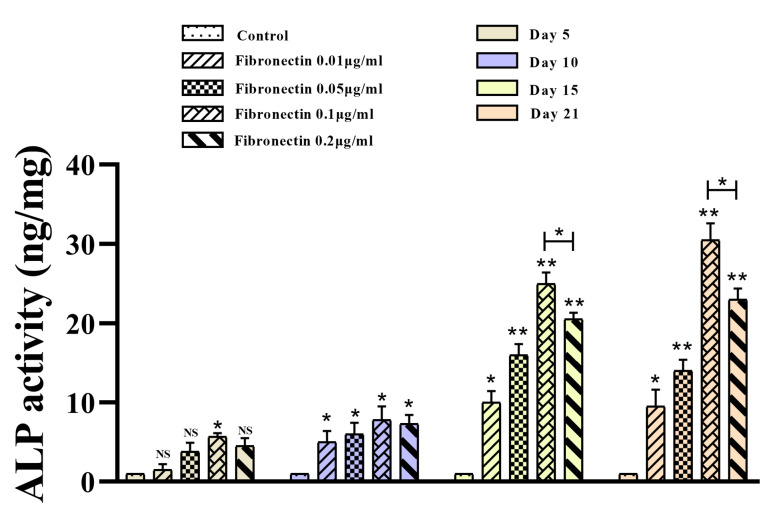
**ALP activity of BMMSCs after osteogenic differentiation in presence of fibronectin.** ALP activity of BMMSCs after was measured in day 5, 10, 15 and 21 in presence of fibronectin (0.01, 0.05, 0.1 and 0.2 μg/ml). (*P* value =0.05)

Based ALP test results, fibronectin 0.01-0.2 μg/mL prominently improved osteoblastic differentiation of BMMSCs compared with the control cells within 5, 10, 15 and 21 days of exposure (*P* < 0.05) (Figure 4). Although the positive effects of the fibronectin 0.1 and 0.2 μg/mL were stronger than fibronectin 0.01 and 0.05 μg/mL, the differentiation potential of fibronectin 0.1 μg/mL was stronger than fibronectin 0.2 μg/mL (*P* < 0.05) (Figure 4). Unsurprisingly, ALP activity of treated BMMSCs within 15 and 21 days of exposure was more prominent than 5 and 10 days of exposure (*P* < 0.05) (Figure 4).

### Fibronectin modified the expression of CDK1, ZBTB16, Twist1, OCN and SP7 genes in BMMSCs

**Figure 5:**
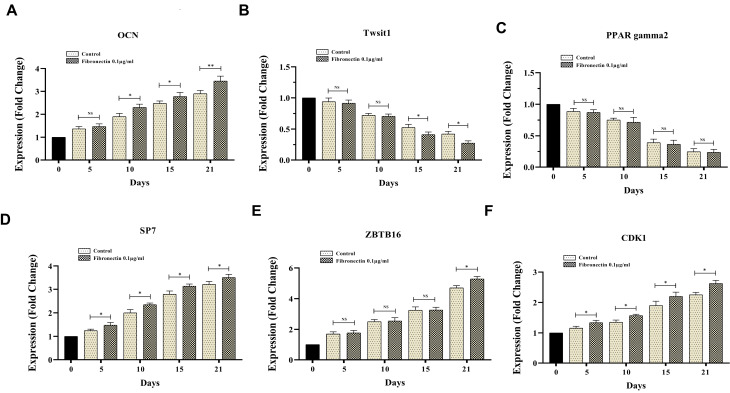
**The effect of 0.1 μg/ml fibronectin on expression of CDK1, ZBTB16, Twist1, OCN and SP7 genes in BMMSCs after osteogenic differentiation**. The expression changes of CDK1, ZBTB16, Twist1, OCN and SP7 genes of BMMSCs after osteogenic differentiation induction was measured in day 5, 10, 15 and 21 in presence of fibronectin (0.1 μg/ml) and compared to control. ( p value =0.05).

RT-PCR results showed that fibronectin 0.1 μg/mL was able to promote OCN expression on days 10, 15 and 21, but not days 5 of ODMSCs compared with the control cells (*P*< 0.05) (Figure 5A). Moreover, fibronectin 0.1 μg/mL attenuated Twist1 expression on days 15 and 21, but not days 5 and 10 of ODMSCs compared with the control cells (*P* < 0.05) (Figure 5B); on the other hand, PPARγ2 expression pattern did not modify following exposure with fibronectin 0.1 μg/mL on any time periods of experiment compared with the control cells (*P* < 0.05) (Figure 5C). Also, results demonstrated fibronectin 0.1 μg/mL positive impacts on SP7 and CDK1 gene expression on days 5, 10, 15 and 21 of ODMSCs compared with the control cells (*P* < 0.05) (Figure 5D, F). But, fibronectin 0.1 μg/mL had the potential to significantly promote ZBTB16 gene expression compared with the control cells only on days 21 of exposure (*P* < 0.05) (Figure 5E).

## Discussion

Recently, MSCs have acquired much attention as a promising therapeutic option for the treatment of a wide range of disorders and abnormalities. According to a large number of studies, ECM is known to play a pivotal role in adjusting the MSCs biologic and functional properties.^27^ In BM, MSCs reside in a dynamic, specialized microenvironment, known as ‘niche’, which delivers extracellular cues to support stem cell viability and identity.^28,29^ The effect of ECM on MSCs is attributed to physical stimulation, such as elasticity and pressure, as well as biochemical stimulation containing growth factors, RNA, and DNA.^30^ Several studies on the regulatory role of ECM on MSCs activities have indicated that cellular density and matrix elasticity as a biological factor play a central role in MSCs function.^31^ Interestingly, it has been noticed that soft and hard culture media lead to differentiation of cells towards neurons and osteoblasts, respectively;^32^ on the other hand, Xue et al found that matrix elasticity adjust the stem cells activities at low cell density but not at high density.^33^ In high cell density, MSCs activities are not modified by the modification in elasticity as cell-to-cell communication defeats cell-matrix communication.^34^ Other types of investigations on the constant matrix and cell density have examined biomaterial’s effects on MSCs biology and functions.^35-37^ Somaiah et al showed that type I collagen improves osteogenic differentiation and proliferation of MSCs through targeting of signaling pathways involved in cell proliferation and commitment.^38^ Linsley et al found that fibronectin, type I collagen, and fibrinogen had the potential to ameliorate growth and osteogenic differentiation of MSCs in the 2D cell culture system.^39^ In this study, we investigated the effect of series concentration of fibronectin on BMMSCs proliferation, differentiation, apoptosis and differentiation involved genes expression levels in a three-dimensional medium. We observed that enhancement of the BMMSCs proliferation and differentiation into the osteoblast takes place up to certain concentration fibronectin (fibronectin 0.1 µg/mL), after which further increase in the concentration of fibronectin attenuated its synergistic effect on BMMSCs pivotal activities. The results of several studies in which fibronectin improved BMMSCs growth and differentiation were consistent with our findings; however, our results were different in the highest concentration. Based on reports, in two-dimensional and three-dimensional media, fibronectin-binding is facilitated by its binding to integrin on cell surface leads to rising proliferation via activation of PI3K/Akt signaling pathway.^40^ Moreover, during osteogenic differentiation, fibronectin triggers a signaling cascade by activating ERK1, 2, which induces RUNX2 signaling pathway.^41,42^ In this study, we evaluated expression levels of some genes, including ZBTB16, Twist1, OCN, SP7, PPARγ2, and CDK1, which adjust osteogenic differentiation of the MSCs, negatively or positively.

ZBTB16 is necessary for the expression of the osteogenic transcription factor RUNX2 and has a significant role in the osteogenic differentiation of dental follicle cells.^43^ Examinations have demonstrated that RUNX2 regulates downstream genes involved in determining of the osteoblast cells phenotype and regulate the expression of OCN and SP7.^44^ Another assessed transcription factor, Twist1 involved in adjusting of the progression and osteogenesis processes, has a notable inhibitory effect on the MSCs osteogenic differentiation.^45^ Moreover, it can negatively adjust the osteoblastic differentiation of the human periodontal ligament cells.^46^ Based on molecular investigations, TWIST1 suppresses RUNX2 expression through directly connecting to an E-box sited at -820 bp of the RUNX2 promoter region and repressing its activity.^47^ Yongkun Wei et al. described that CDK1-dependent phosphorylation of Enhancer of zeste homolog 2 (EZH2) reduced methylation of H3K27 and improves osteogenic differentiation of human MSCs.^48^

Considering the results obtained in this study, fibronectin was able to promote osteogenic differentiation of BMMSCs through enhancing of the OCN, SP7, ZBTB16, and CDK1, and reducting of Twist1 expression compared with the control cells. It seems that CDK1 upregulation leads to increased methylation of the EZH2, which in turn augments the osteogenic differentiation potential of the MSCs. We believe that Twist downregulation in association with ZBTB16 upregulation led to the promotion of RUNX2 expression and its target genes, such as OCN and SP7, which finally ameliorated osteogenic differentiation of the MSCs following exposure with fibronectin. Albeit, it seems signals triggered by fibronectin into the MSCs are insufficient to these cells complete their osteogenic differentiation, and therefore synergistic signals from the surrounding environment are required. Mohamadyar-Toupkanlou et al have indicated that calcium deposition does not occur due to mere osteogenic differentiation of MSCs in the presence of fibronectin, but takes place when combined with hydroxylapatite nanoparticles.^49^ In this research, sodium alginate microcapsule as a synergistic agent led to the completion of BMMSCs osteogenic differentiation and calcium deposition. Since fibronectin is not a protein specific to ECM, we believe that the effect of fibronectin on the function of BMMSCs is related to the concentration of fibronectin. We believe that at high concentrations of fibronectin, the proximity of these molecules causes the connection between these molecules and thereby conceals their cell-binding site, which, in turn, attenuated its synergistic effect on the MSCs pivotal activities, such as proliferation and differentiation.

## Conclusion

According to the wide range of studies, the cells should make contact with other cells and ECM to become biologically functional. Moreover, because of the absence of toxicity and high bioavailability, sodium alginate microcapsules possess a noticeable potential to encapsulate MSCs. Currently, it is expected to take a significant step forward into the optimization of MSCs growth and osteogenic differentiation in vitro aiming to treat bone-related diseases by promotion of the synergistic influences of fibronectin on the BMMSC activities. Based on the results, fibronectin had the potential to improve osteogenic differentiation of BMMSCs through modification of OCN, SP7, ZBTB16, CDK1 and Twist1 expression in dose dependent manner. We found that a further increase in its concentration (up to 0.2 µg/mL) attenuated the synergistic effect of fibronectin on BMMSCs proliferation and differentiation. Our results could be used to the optimization of BMMSC proliferation and differentiation in the laboratory environment as well as the treatment of bone disorders.

## Ethical Issue

No animal or human studies were carried out by the authors.

## Conflict of Interest

The authors declare that there are no conflicts of interest.

## Acknowledgments

We highly appreciate the help of stem cell and Tissue Engineering Research Laboratory, Sahand University of Technology, Tabriz in providing laboratory facilities for this research. We express our appreciation to Tabriz University of Medical Sciences, Tabriz, Iran for financial support of this research. There is no conflict of interest in this study.
